# Is There a Difference in the Diagnosis and Prognosis of Local Recurrence between Autologous Tissue and Implant-Based Breast Reconstruction?

**DOI:** 10.1155/2022/9029528

**Published:** 2022-04-11

**Authors:** Kyunghyun Min, Hyun Ho Han, Eun Key Kim, Sae Byul Lee, Jisun Kim, Il Yong Chung, Hee Jeong Kim, Beom Seok Ko, Jong Won Lee, Byung Ho Son, Sei Hyun Ahn, Jin Sup Eom

**Affiliations:** ^1^Department of Plastic and Reconstructive Surgery, Hanyang University College of Medicine, Seoul, Republic of Korea; ^2^Department of Plastic Surgery, Asan Medical Center, University of Ulsan, College of Medicine, Ulsan, Republic of Korea

## Abstract

**Introduction:**

Breast reconstruction has become common after total mastectomy; however, certain types of breast reconstruction may be associated with delayed local recurrence or poor survival. Here, we investigated whether there are differences in the diagnosis and prognosis of local recurrence between autologous reconstruction and implant reconstruction.

**Materials and Methods:**

A retrospective analysis was performed on patients undergoing breast cancer surgery with autologous tissue or immediate implant reconstruction in a single center (January 2003-December 2017). Patient data including the period from cancer surgery to local recurrence diagnosis, tumor size at the time of recurrence, and survival time after cancer surgery and recurrence detection were analyzed.

**Results:**

There was a significant difference (*p* = 0.021) in the time from surgery to recurrence between the autologous tissue (1,246 days) and implant (909 days) groups. Recurrence tumor size did not differ (autologous: 1.00 cm^2^ vs. implant: 0.90 cm^2^; *p* = 0.813). Survival time after surgery (*p* = 0.63) and recurrence detection (*p* = 0.74) did not statistically significant.

**Conclusions:**

Statistical difference in the detection time was observed between autologous tissue and implant group. On the other hand, there is no difference in recurrence tumor size or survival time. A further study is necessary to identify the different detection time of local recurrence.

## 1. Introduction

According to American Cancer Society data, 252,710 cases of invasive breast cancer were diagnosed in 2017. Furthermore, 63,410 cases of carcinoma *in situ* detection and 101,657 cases of breast reconstruction were performed in 2018 [1]. These statistics reveal an increase in the incidence by more than 20,000 cases relative to that reported in 2000 [1]. 

Postmastectomy breast reconstruction offers the option of reconstruction with either autologous tissue or an implant. In cases undergoing autologous tissue reconstruction, the quality of the material is similar to natural glandular tissue, and flap tailoring is possible considering the patient's breast shape. Moreover, there is no immune response such as capsule formation, and there is a reduced likelihood of infection. However, the method usually disrupts the normal anatomy; leaves long, undesirable scars on the donor site; and is more damaging in cases of reconstruction failure.

Conversely, the advantages of implants include simple surgical procedures, reconstruction without damaging other normal tissues, and rapid recovery. However, implant malposition may occur. Furthermore, implant products cannot be customized for each patient; therefore, asymmetry occurs more frequently than autologous reconstruction. Unpredictable capsular contracture, seroma, and breast implant–associated anaplastic large-cell lymphoma are also well-known potential risks [2, 3]. Moreover, postoperative complications such as wound dehiscence and subsequent exposure can be increased by postoperative radiotherapy [4].

As each reconstruction method has distinct advantages and disadvantages, reconstruction choices can differ according to the doctor's preferences, medical insurance system, and the patient's socioeconomic status and decision [5]. However, it remains controversial whether to consider implants or autologous reconstruction first.

**Figure 1:**
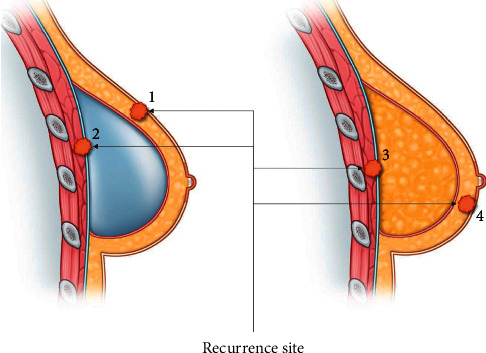
Site of local recurrence detection. 1. Implant insertion can cause the mass to be touched more easily on the surface. 2. However, when screening modalities such as ultrasound are used, it may be difficult to detect recurring masses in the bottom layer beneath the implant. 3, 4. Autologous tissue below the mastectomy skin flap may interfere with the detection of a newly formed nodule, and fat necrosis can confuse the detection and discrimination of recurrent cancer.

Furthermore, a more important consideration to adopt in choosing the modality of reconstruction is whether the reconstruction itself influences patient prognosis after cancer treatment. Autologous tissue below the mastectomy skin flap may interfere with the detection of newly formed nodules, and fat necrosis can confuse the discriminating mode used to detect recurrent cancer [6, 7]. Recurrence after implant insertion can cause the mass to be touched more easily on the surface. However, when using screening modalities such as ultrasound, it may be challenging to detect recurring masses beneath the implant (Figure 1) [8]. In this regard, there is a concern that either reconstruction may be disadvantageous in allowing precise detection of the local recurrence in the breast.

The purpose of this study was to determine whether there is a difference in the diagnosis, treatment process, and prognosis of local recurrence between autologous tissue reconstruction and implant reconstruction.

## 2. Materials and Methods

A retrospective analysis was performed on patients undergoing breast cancer surgery in a single center between January 1, 2003, and December 31, 2017, after institutional review board approval (IRB 2020-0035). All eligible patients were classified as those undergoing reconstruction by autologous tissue or immediate implantation, respectively. Among them, only those with local recurrence were further included in this study. Patients who received breast-preserving surgery, breast reconstruction before the diagnosis of breast cancer, or breast reconstruction using both autologous tissue and implants were excluded from data analysis. Additionally, patients who had cancer stage IIIB or higher, those with a serious history (e.g., primary malignancy in other sites, severe cardiac/pulmonary disease), or those who died owing to a reason other than the recurrence of breast cancer were excluded from the study.

### 2.1. Inclusion Criteria for Local Recurrence

**Figure 2:**
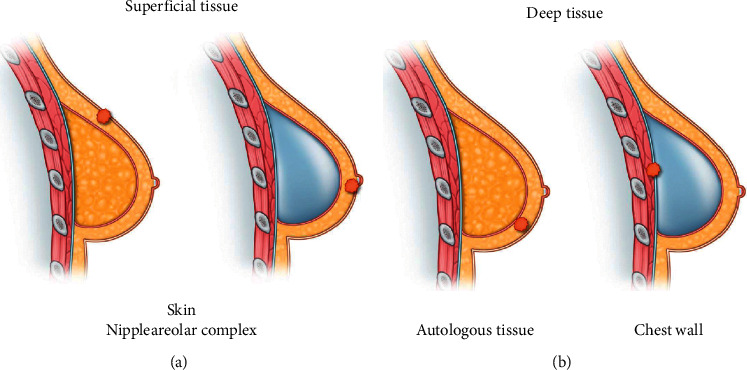
Type of local recurrence. (a) Superficial tissue recurrence; (b) deep tissue recurrence. Cases of local recurrence with any regional recurrence or distant metastasis were excluded.

We included patients diagnosed with local recurrence that could be detected superficially in the skin and nipple-areolar complex or in deep tissues such as the subcutaneous layer or chest wall of mastectomy (Figure 2). Therefore, patients with regional recurrences such as axillary lymph node metastasis or distant metastasis were excluded from the present study regardless of the presence of local recurrence. This exclusion was conducted because regional and distant metastases can be found in areas that are far apart, independently of the reconstruction. Moreover, when any metastasis was combined with local recurrence, the sequence of recurrence could not be clearly identified.

### 2.2. Collecting Data

Demographics, such as age, body mass index (BMI), history of diabetes, hypertension, and smoking, were assessed. Furthermore, the stage, hormonal status (estrogen receptor (ER), progesterone receptor (PR), and Her2), and preoperative/preoperative treatment of cancer diagnosed at the time of surgery were reviewed, and the tumor size and location depth at the time of the initial recurrence were noted. We investigated whether outpatients suspected of recurrence had scheduled visits or unplanned visits. Finally, we compared periods of time from breast cancer surgery to recurrence diagnosis, salvage of reconstructive reconstruction after recurrence, and breast reshaping in salvaged cases between the two reconstructions. The definition of salvage in the autologous group was that more than 50% of the reconstructed tissue was preserved.

### 2.3. Statistical Analysis

The Statistical Package for the Social Sciences version 26.0 software (IBM Co., Armonk, NY) was used to confirm the statistical significance of data collected from the groups. Chi-squared test, Student's *t*-test, and Mann–Whitney *U*-test were employed for comparing continuous and categorical variables between two groups. A linear regression analysis was conducted to clarify the variables, which affects the cancer surgery to recurrence detection period. Log-transformation was necessary because the data were skewed to the right side. The fitness was tested through the Kolmogorov–Smirnov test, Cramer–von Mises test, and Anderson–Darling test to examine whether the cancer surgery to recurrence detection rate period, which appeared to be similar to the gamma distribution, was affected by the hormone receptors and Her2. The effect of hormone receptor and Her2 was also confirmed using a linear regression model. In addition, a Kaplan–Meier curve was used to compare recurrence with the time of death and cancer surgery with the time of death. Statistical significance was confirmed using the log-rank test.

## 3. Results

Over 15 years, a total of 2,361 autologous tissue reconstructions and 551 immediate implants were performed. In total, 93 (3.94%) patients were diagnosed as having local recurrence in the autologous tissue group and 25 (4.54%) patients were diagnosed with the same in the immediate implant group (*p*=0.521). The mean age of the autologous group (40.03 ± 7.59 years) was older than that of the immediate implant group (35.12 ± 6.75 years; *p*=0.004). In addition, there was a difference in the mean BMI (autologous tissue: 22.68 ± 2.69 kg/m^2^ vs. immediate implant: 21.14 ± 2.80 kg/m^2^; *p*=0.013). A history of diabetes, hypertension, and smoking was not different between the two groups.

The cancer stage distribution of patients was not statistically significant between the autologous tissue and implant groups (*p*=0.261). Neither the history of neoadjuvant chemotherapy nor adjuvant chemo- and radiotherapy was not significant (Table 1).

Comparing the periods from breast cancer surgery to recurrence diagnosis showed that the autologous tissue group experienced a longer period at 1,246 (742–1,820) days, while the immediate implant group exhibited the shorter period at 909 (384–1,231) days, with statistical significance found between the two groups (*p*=0.021).

The detected tumor size at the time of recurrence was 1.09 cm^2^ (0.94–1.23) in the autologous tissue group and 1.11 cm^2^ (0.82–1.4) in the immediate implant group, indicating that there was no difference in this regard between the two groups (*p*=0.868).

In the classification of the location of recurrence, implant patients were diagnosed with a higher rate of deep tissue recurrence compared to that of the autologous group (implant: 9 of 25, 36% vs. autologous tissue: 24 of 93, 25.8%). However, there was no statistical significance (*p*=0.449).

Considering the type of outpatient visits in those cases where the patient was diagnosed with recurrence, the immediate implant group had a higher rate of unplanned visits (13 patients; 52.0%); however, no statistical significance was found between the two groups (*p*=0.203).

Three patients diagnosed with local recurrence underwent a local advancement flap after surgical removal, while one patient underwent reconstruction with a mini latissimus dorsi musculocutaneous flap. Initially, all patients underwent reconstruction using autologous tissue. Failure to salvage the existing autologous reconstruction occurred in no patient. In one patient who underwent reconstruction with an implant, the device was removed and replaced with an expander because of the difficulty of salvage (Table 2). The rest of both group patients underwent simple wide excision with primary closure.

According to linear regression analyses, for the period of time between cancer surgery and the detection of recurrence, the type of reconstruction, patient age, and reception of neoadjuvant chemotherapy were significant in the univariate analysis, while only reception of neoadjuvant chemotherapy was statistically significant in the multivariable analysis (best point estimate: −0.553; 95% CI −0.928 –−0.178; *p*=0.001, Table 3).

Based on ER/PR/Her2 expression, it was classified into four subgroups: Group 1: ER (+), PR (any), and Her2 (+); Group 2: ER (+), PR (any), and Her2 (-); Group 3: ER (-), PR (-), and Her2 (+); and Group 4: ER (-), PR (-), and Her2 (-). The cancer surgery to recurrence detection period of Group 2 was the longest in both autologous and implant groups. However, no statistical significance was observed between the two groups (autologous group: 1,471 (1,003–1,827) days and implant group: 1,351.5 (1,127–1,707) days; *p*=0.7128). By contrast, Group 4, known as triple-negative type, showed the shortest period from the cancer surgery to recurrence detection (autologous group: 743 (225–1,246) days and implant group: 535.5 (194–877) days; *p*=0.7728). However, no statistical significance was observed when comparing the four groups of cancer surgery to the recurrence detection period (autologous group: *p*=0.0877 and implant group: *p*=0.2812, Table 4).

As a result of exploratively confirming the effect of the interaction between the autologous group and ER, PR, and Her2 on the cancer surgery to the recurrence detection period using a linear regression model, the period was shorter in the case of the implant group than in the case of the autologous group (estimate: −0.3615 and standard error: 0.1501; *p*=0.0177). A relatively long period was confirmed in the case of ER-positive breast cancer, but the statistical significance was on the boundary line (estimate: 0.4242 and standard error: 0.2198; *p*=0.0562). PR and Her2 did not show statistical significance.

**Figure 3:**
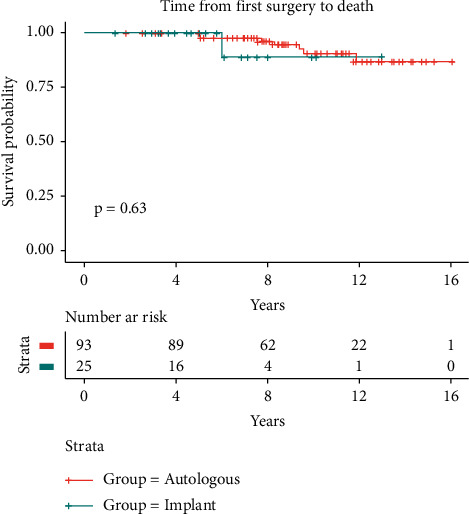
Survival analysis: cancer surgery to the time of death.

**Figure 4:**
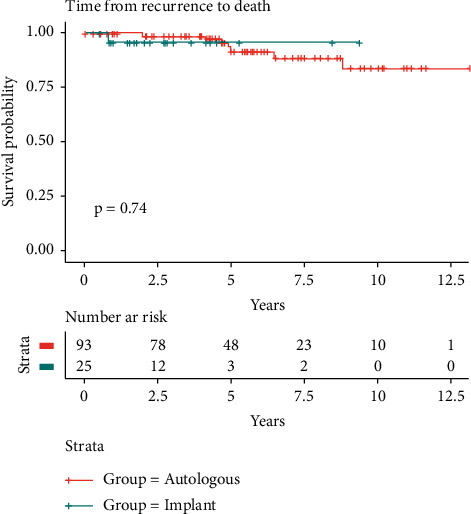
Survival analysis: local recurrence to the time of death.

Finally, the survival times after surgery and after the detection of recurrence, respectively, were not significantly different (time from first surgery to death: *p*=0.63 vs. time from recurrence to death: *p* = 0.74*p*=0.74) in the two groups (Figures 3 and 4).

## 4. Discussion

Because both advantages and disadvantages, although different depending upon the choice of the breast reconstruction method, remain in existence overall, doctors must discuss with individual patients the reconstruction method to be used in each case. Results of previous studies on breast cancer recurrence and survival rates have supported that reconstruction does not increase the recurrence of breast cancer and cannot affect the survival rate of these patients [9–20]. However, some surgeons still have concerns that autologous tissues or implants may act as obstacles in the diagnosis of the local recurrence of breast cancer [21].

Siotos et al. studied differences in survival rates between reconstructed and nonreconstructed cases among 1,517 patients with breast cancer. According to their study, there was a 20% higher overall survival benefit in the reconstruction group [22]. Factors contributing to survival, such as differences in race, income, and socioeconomic status, and the varying effects of instruction and counseling on reconstruction outcomes among those who might appreciate such (i.e., those with a higher education level) versus those who may not are not yet fully understood.

Kanchwala et al. studied 41 patients with locoregional recurrence. The time required to pinpoint recurrence did not differ between the immediate implant and autologous tissue groups. The average tumor size in patients with recurrent cancer was 1.5 cm in the immediate implant group and 2.9 cm in the autologous tissue group, with the immediate implant group showing nearly double the rate of index reconstruction loss [23]. However, it was hard to elucidate the incidence of locoregional recurrence because the authors did not report the total number of mastectomy and reconstruction procedures. Furthermore, when assessing reconstruction salvage, the implants can be clearly distinguished because device explantation is considered as a failure, while in cases of autologous reconstruction, the salvage definition might be more vague, which potentially affected the credibility of their data.

In this study, attempting to assess the local recurrence is consistent with our research, we clarified the definition of salvage in the autologous group. Furthermore, medical treatment was conducted through a single and equivalent public insurance system. These can bring us the benefit of data accuracy because these kinds of social systems automatically control variables and factors that may bias statistics.

According to the result of retrospective observation, the period from cancer surgery to recurrence detection was shorter in the immediate implant group. Meanwhile, based on the linear regression test, only neoadjuvant chemotherapy was found to have a negative factor, which affects the time from initial breast cancer resection to the diagnosis of local recurrence. Considering that the general indications for neoadjuvant chemotherapy are locally advanced breast cancer or clinically node-positive breast cancer, [24] the result may be a factor affecting the detection period in two groups in which the patient's distribution of neoadjuvant chemotherapy was different.

Another factor that can have a significant effect on cancer surgery to local recurrence detection period is the hormone receptor and Her2 expression. According to previous studies, the prognosis is best for ER-positive, any PR, and Her2-negative breast cancer and poor for triple-negative breast cancer [25, 26]. As a result of the classification by hormone receptor and Her2 expression, results similar to those of previous studies were obtained in absolute values, although statistical significance could not be observed. When the hormone receptor and Her2 distributions in the autologous and implant group patients were summarized, 39.78 percent of patients were ER-positive and Her2-negative in the autologous group; however, only 24 percent of patients were diagnosed ER-positive and Her2-negative in the implant group, whereas 11 (44%) patients were ER- and Her2-positive. A total of 3 (3.23%) patients were triple-negative breast cancer patients in the autologous group; however, the percentage of triple-negative breast cancer was more than twice as much in the case of the implant group (2 (8%) patients, *p*=0.636). These data also indicate that local recurrence occurred later in the autologous group than in the implant group.

However, the factors contributing to the difference in the detection period between the two groups are various, such as the staging differences and pure detection issues. Therefore, it is unreasonable to conclude that autologous reconstruction has a more disturbing effect on local recurrence detection than implant reconstruction based on the results of this study. Large samples and prospective studies are necessary to identify whether and why autologous reconstruction and implant reconstruction have the different detection period after local recurrence.

Meanwhile, the survival rate is the most important factor to distinguish the difference in local recurrence prognosis. In the study, the postoperative survival period and survival period after recurrence detection were not statistically significantly different between the two groups. Contrary to the concerns of some surgeons, there was no difference in local recurrence findings between autologous tissue reconstruction and implants or any statistical difference in terms of survival.

The incidence of local recurrence of breast cancer in this study was less than 5%; therefore, the study population may be too small in this regard to draw certain conclusions from in this study. However, the present study drew conclusions based on the accumulation of 15 years of data. Furthermore, unlike in previous papers of locoregional recurrence that includes the recurrence of lymph nodes or distant metastasis, our study clarified the definition of local recurrence while excluding lymph node recurrence or metastasis. And distant metastasis cases were also excluded for this same reason. Therefore, the present study offers good practical evidence regarding the direct effect of the two reconstruction methods on the diagnosis of local recurrence.

Because the study was conducted with patients belonging to a single race who benefited from the national healthcare service, the environmental factor was automatically controlled to increase the reliability of the study. Altogether, this study followed a systematic approach to determine whether there exist variations in the recurrence detection and survival period of patients when treated with different breast reconstruction methods. It is expected that this will be a reasonable basis for the assumption that breast implant or autologous tissue reconstruction does not cause harmful effects in the diagnosis and treatment of local recurrence.

There are other limitations to this study. First, the immediate reconstruction method involving the use of implants was initiated in 2008, resulting in a relatively small number of reconstructions and a short follow-up period. Second, although no significance was observed in both univariate and multivariate analyses, patient age and BMI values were different between the two groups. This may be a limitation because of the small number of patients who only developed local recurrence after breast cancer surgery. We expect that more accurate results will be obtained if we examine the matched patients throughout a longer follow-up period.

## 5. Conclusions

According to the retrospective observation, longer period of local recurrence detection was observed in autologous reconstruction group. On the other hand, there was no statistical significance in tumor size at the time of recurrence and survival rate between the two groups. Further studies are required whether autologous and implant groups do not have a difference in the diagnosis of local recurrence and the survival prognosis.

## Figures and Tables

**Table 1:** Patients' demographics, stage, and pre-/postoperative therapy.

	Autologous	Implant	*p* value
All patients	2361	551	
Local recurrence (%)	93 (3.94)	25 (4.54)	0.521
Age (mean (SD))	40.03 (7.59)	35.12 (6.75)	0.004
Age (median [IQR])	40.00 [35.00, 44.00]	33.00 [30.00, 36.00]	0.002
Stage (%)			0.261
0	13 (14.0)	2 (8.0)	
1	44 (47.3)	9 (36.0)	
2	33 (35.5)	14 (56.0)	
3	3 (3.2)	0 ( 0.0)	
ER = 1 (%)	71 (76.3)	17 (68.0)	0.554
PR = 1 (%)	65 (69.9)	13 (52.0)	0.150
HER = (%)	52 (55.9)	17 (68.0)	0.390
BMI (mean (SD))	22.68 (2.69)	21.14 (2.80)	0.013
BMI (median [IQR])	22.50 [20.83, 23.85]	20.69 [19.51, 22.59]	0.007
DM = 1 (%)	1 (1.1)	0 (0.0)	1.000
HTN = 1 (%)	3 (3.2)	0 (0.0)	0.846
Smoking = 1 (%)	5 (5.4)	1 (4.0)	1.000
NeoCTx. = 1 (%)	7 (7.5)	5 (20.0)	0.145
RTx. = 1 (%)	6 (6.5)	1 (4.0)	1.000
Post-CTx. = 1 (%)	30 (32.3)	8 (32.0)	1.000
Hormone Tx. = 1 (%)	62 (66.7)	14 (56.0)	0.451
Herceptin = 1 (%)	7 (7.5)	5 (20.0)	0.145

SD, standard deviation; IQR, interquartile range; NeoCTx., neoadjuvant chemotherapy; RTx., adjuvant radiotherapy; Post-CTx., adjuvant chemotherapy; Hormone Tx., hormone therapy.

**Table 2:** Local recurrence profiles.

	Autologous	Implant	*p* value
Cancer surgery to recurrence detection	1246	909	0.021
(days, median [IQR])	[742.00, 1820.00]	[384.00, 1231.00]	
Tumor size (cm^2^, mean (95% CI))	1.09 (0.94, 1.23)	1.11 (0.82, 1.4)	0.868
Tumor size (cm^2^, median [IQR])	1.00 [0.70, 1.20]	0.90 [0.60, 1.20]	0.813
Deep tissue recurrence (%)	24 (25.8)	9 (36.0)	0.449
95% CI of percentage	17.3, 35.9	18.0, 57.5	
The number of unplanned visit	33	13	0.203
(% (95% CI))	35.5 (17.3, 35.9)	52.0 (18.0, 57.5)	
Reconstruction after recurrent breast cancer operation (%)	4 (4.30)	1 (4)	1.000
Failed to salvage of previous reconstruction (%)	0 (0)	1 (4)	0.052

IQR, Interquartile range; CI, confidence interval.

**Table 3:** Period between cancer surgery to the detection of recurrence: univariate and multivariable analyses.

	Univariate				Multivariable			
	Beta point estimate	95% CI		*p* value	Beta point estimate	95% CI		*p* value
		LB	UB			LB	UB	
Group (reference: Implant)	−0.411	−0.749	−0.074	0.017	−0.286	−0.625	0.054	0.098
Age	0.018	0.000	0.037	0.047	0.009	–0.010	0.028	0.373
BMI	0.005	–0.046	0.056	0.854	−0.011	−0.062	0.040	0.670
NeoCTx.	–0.707	–1.045	–0.369	<0.001	–0.553	–0.928	–0.178	0.001

CI, confidence interval; LB, lower bound of uncertainty interval; UB, upper bound of uncertainty interval; NeoCTx., neoadjuvant chemotherapy.

**Table 4:** Subgroup analysis by ER, PR, and Her2 expression.

Subgroup	ER (+), PR (+ or −), Her2 (+)					ER (+), PR (+ or −), Her2 (-)					ER (-), PR (-), Her2 (+)					ER (-), PR (-), Her2 (-)				
	Autologous		Implant			Autologous		Implant			Autologous		Implant			Autologous		Implant		
	*n* = 34		*n* = 11		*p* value	*n* = 37		*n* = 6		*p* value	*n* = 18		*n* = 6		*p* value	*n* = 3		*n* = 2		*p* value
Age (mean (SD))	40.21	7.73	34.18	7.29	0.0279	40.14	7.96	36.00	4.15	0.2232	40.56	7.22	36.33	9.46	0.2624	36.33	5.03	34.00	1.41	0.5849
Age (median [IQR])	40	[35.00, 46.00]	31	[29.00, 36.00]	0.0139	40	[36.00, 44.00]	34.50	[33.00, 40.00]	0.1654	40.00	[34.00, 48.00]	33.50	[30.00, 41.00]	0.1919	37.00	[31.00, 41.00]	34.00	[33.00, 35.00]	0.7728
Stage (%)					0.5618					0.5871					0.8175					1.0000
0	4	11.76	2	18.18		6	16.22	0	0.00		3	16.67	0	0.00		1	33.33	0	0.00	
1	15	44.12	3	27.27		16	43.24	2	33.33		11	61.11	4	66.67		2	66.67	2	100.00	
2	12	35.29	6	54.55		15	40.54	4	66.67		4	22.22	2	33.33						
3	3	8.82	0	0.00																
BMI (mean (SD))	22.7	2.2	21.81	2.42	0.2624	22.11	2.27	19.31	2.67	0.0091	23.85	3.96	21.76	3.50	0.2627	22.00	2.75	21.02	2.14	0.7034
BMI (median [IQR])	22.58	[21.19, 23.81]	21.2	[19.69, 23.07]	0.2399	22.11	[20.51, 23.28]	19.84	[17.59, 20.69]	0.0217	23.42	[20.08, 26.67]	21.57	[18.81, 21.63]	0.1936	21.34	[19.64, 25.02]	21.02	[19.51, 22.53]	0.7728
DM = 1 (%)	0	0.00	0	0.00	-	1	2.70	0	0.00	1.0000	0	0.00	0	0.00	-	0	0.00	0	0.00	-
HTN = 1 (%)	1	2.94	0	0.00	1.0000	2	5.41	0	0.00	1.0000	0	0.00	0	0.00	-	0	0.00	0	0.00	-
Smoking = 1 (%)	3	8.82	0	0.00	1.0000	1	2.70	1	16.67	0.2625	1	5.56	0	0.00	1.0000	0	0.00	0	0.00	-
Tumor size (cm^2^, mean (95% CI))	0.99	(0.80, 1.17)	1.1	(0.59, 1.61)	0.5817	1.14	(0.85, 1.46)	0.75	(0.45, 1.05)	0.0408	1.08	(0.63, 2.37)	1.50	(0.63, 2.37)	0.2543	1.30	(−0.21, 2.80)	1.10	(−3.98, 6.18)	0.7369
Tumor size (cm^2^_,_ median [IQR])	0.95	[0.6, 1.2]	0.9	[0.60, 1.40]	0.905	0.90	[0.70, 1.20]	0.75	[0.50, 1.00]	0.1931	1.10	[0.60, 1.50]	1.10	[0.90, 2.40]	0.4222	1.00	[0.90, 2.00]	1.10	[0.70, 1.50]	0.7728
Cancer surgery to recurrence detection	1242.5	[762, 2378]	818	[369, 1172]	0.0314	1471	[1003, 1827]	1351.5	[1127, 1707]	0.7128	848	[687, 1330]	900	[499, 1342]	0.8676	743	[225, 1246]	535.5	[194, 877]	0.7728
(days, median [IQR])																				

## Data Availability

The data used to support the findings of this study are included within the article.

## References

[1] Rose J., Puckett Y. (2020). *Breast Reconstruction Free Flaps*.

[2] Dashevsky B. Z., Gallagher K. M., Grabenstetter A. (2019). Breast implant‐associated anaplastic large cell lymphoma: clinical and imaging findings at a large US cancer center. *Breast Journal*.

[3] Leberfinger A. N., Behar B. J., Williams N. C. (2017). Breast implant-associated anaplastic large cell lymphoma. *JAMA Surgery*.

[4] Yun J. H., Diaz R., Orman A. G. (2018). Breast reconstruction and radiation therapy. *Cancer Control: Journal of the Moffitt Cancer Center*.

[5] Sheckter C. C., Panchal H. J., Razdan S. N. (2018). The influence of physician payments on the method of breast reconstruction. *Plastic and Reconstructive Surgery*.

[6] Hsu W., Sheen-Chen S.-M., Eng H.-L., Ko S.-F. (2008). Mammographic microcalcification in an autogenously reconstructed breast simulating recurrent carcinoma. *Tumori Journal*.

[7] Eidelman Y., Liebling R. W., Buchbinder S., Strauch B., Goldstein R. D. (1998). Mammography in the evaluation of masses in breasts reconstructed with TRAM flaps. *Annals of Plastic Surgery*.

[8] Juanpere S., Perez E., Huc O., Motos N., Pont J., Pedraza S. (2011). Imaging of breast implants-a pictorial review. *Insights into Imaging*.

[9] McCarthy C. M., Pusic A. L., Sclafani L. (2008). Breast cancer recurrence following prosthetic, postmastectomy reconstruction: incidence, detection, and treatment. *Plastic and Reconstructive Surgery*.

[10] Petit J. Y., Gentilini O., Rotmensz N. (2008). Oncological results of immediate breast reconstruction: long term follow-up of a large series at a single institution. *Breast Cancer Research and Treatment*.

[11] Bezuhly M., Temple C., Sigurdson L. J., Davis R. B., Flowerdew G., Cook E. F. (2009). Immediate postmastectomy reconstruction is associated with improved breast cancer-specific survival. *Cancer*.

[12] Nedumpara T., Jonker L., Williams M. R. (2011). Impact of immediate breast reconstruction on breast cancer recurrence and survival. *The Breast*.

[13] Reddy S., Colakoglu S., Curtis M. S. (2011). Breast cancer recurrence following postmastectomy reconstruction compared to mastectomy with no reconstruction. *Annals of Plastic Surgery*.

[14] Gieni M., Avram R., Dickson L. (2012). Local breast cancer recurrence after mastectomy and immediate breast reconstruction for invasive cancer: a meta-analysis. *The Breast*.

[15] Lee T. J., Hur W. J., Kim E. K., Ahn S. H. (2012). Outcome of management of local recurrence after immediate transverse rectus abdominis myocutaneous flap breast reconstruction. *Archives of Plastic Surgery*.

[16] Patterson S. G., Teller P., Iyengar R. (2012). Locoregional recurrence after mastectomy with immediate transverse rectus abdominis myocutaneous (TRAM) flap reconstruction. *Annals of Surgical Oncology*.

[17] Platt J., Baxter N. N., McLaughlin J., Semple J. L. (2015). Does breast reconstruction after mastectomy for breast cancer affect overall survival? Long-term follow-up of a retrospective population-based cohort. *Plastic and Reconstructive Surgery*.

[18] Ilonzo N., Tsang A., Tsantes S., Estabrook A., Thu Ma A. M. (2017). Breast reconstruction after mastectomy: a ten-year analysis of trends and immediate postoperative outcomes. *The Breast*.

[19] Ryu J. M., Paik H.-J., Park S. (2017). Oncologic outcomes after immediate breast reconstruction following total mastectomy in patients with breast cancer: a matched case-control study. *Journal of Breast Cancer*.

[20] Zhang P., Li C.-Z., Wu C.-T. (2017). Comparison of immediate breast reconstruction after mastectomy and mastectomy alone for breast cancer: a meta-analysis. *European Journal of Surgical Oncology*.

[21] Coroneos C. J., Roth-Albin K., Rai A. S. (2017). Barriers, beliefs and practice patterns for breast cancer reconstruction: a provincial survey. *The Breast*.

[22] Siotos C., Naska A., Bello R. J. (2019). Survival and disease recurrence rates among breast cancer patients following mastectomy with or without breast reconstruction. *Plastic and Reconstructive Surgery*.

[23] Mirzabeigi M. N., Rhemtulla I. A., McDonald E. S. (2019). Locoregional cancer recurrence after breast reconstruction. *Plastic and Reconstructive Surgery*.

[24] Masood S. (2016). Neoadjuvant chemotherapy in breast cancers. *Women’s Health*.

[25] Harbeck N., Gnant M. (2017). Breast cancer. *The Lancet*.

[26] Waks A. G., Winer E. P. (2019). Breast cancer treatment. *JAMA*.

[27] https://www.researchsquare.com/article/rs-172420/v1.

